# Digital Intervention Barriers Scale–7 (DIBS-7): Development, Evaluation, and Preliminary Validation

**DOI:** 10.2196/40509

**Published:** 2023-04-06

**Authors:** Giovanni Ramos, Amanda Kay Montoya, Hayley Renee Hammons, Danielle Smith, Denise April Chavira, Leslie Rose Rith-Najarian

**Affiliations:** 1 Department of Psychology University of California, Los Angeles Los Angeles, CA United States; 2 Department of Psychology Harvard University Cambridge, MA United States

**Keywords:** barriers, development, digital mental health intervention, measure, psychometrics, scale, validation

## Abstract

**Background:**

The translation of mental health services into digital formats, deemed digital mental health interventions (DMHIs), has the potential to address long-standing obstacles to accessing care. However, DMHIs have barriers of their own that impact enrollment, adherence, and attrition in these programs. Unlike in traditional face-to-face therapy, there is a paucity of standardized and validated measures of barriers in DMHIs.

**Objective:**

In this study, we describe the preliminary development and evaluation of such a scale, the Digital Intervention Barriers Scale-7 (DIBS-7).

**Methods:**

Following an iterative QUAN → QUAL mixed methods approach, item generation was guided by qualitative analysis of feedback from participants (n=259) who completed a DMHI trial for anxiety and depression and identified barriers related to self-motivation, ease of use, acceptability, and comprehension of tasks. Item refinement was achieved through DMHI expert review. A final item pool was administered to 559 treatment completers (mean age 23.02 years; 438/559, 78.4% female; 374/559, 69.9% racially or ethnically minoritized). Exploratory factor analyses and confirmatory factor analyses were estimated to determine the psychometric properties of the measure. Finally, criterion-related validity was examined by estimating partial correlations between the DIBS-7 mean score and constructs related to treatment engagement in DMHIs.

**Results:**

Statistical analyses estimated a 7-item unidimensional scale with high internal consistency (α=.82, ω=0.89). Preliminary criterion-related validity was supported by significant partial correlations between the DIBS-7 mean score and treatment expectations (pr=–0.25), number of modules with activity (pr=–0.55), number of weekly check-ins (pr=–0.28), and treatment satisfaction (pr=–0.71).

**Conclusions:**

Overall, these results provide preliminary support for the use of the DIBS-7 as a potentially useful short scale for clinicians and researchers interested in measuring an important variable often associated with treatment adherence and outcomes in DMHIs.

## Introduction

Despite the ubiquity of mental disorders worldwide, unmet mental health need exceeds 60% in affluent countries and nearly reaches 90% in low-income countries [1]. Although no single approach will address this treatment gap, the use of technology to deliver care represents a paradigm shift that could address the shortage of mental health professionals, mitigate logistic barriers to service use, and engage individuals in care who may not otherwise seek services [2]. Digital mental health interventions (DMHIs) have been shown to be effective, often leading to similar treatment outcomes as traditional face-to-face interventions [3-5]. Promising results have also been found in marginalized groups, such as racially or ethnically minoritized groups, individuals living in rural communities, persons experiencing homelessness, and sexual- and gender-minoritized individuals [6,7]. Further, DMHI users consistently report high treatment acceptability and satisfaction [8-10].

Despite the promise of DMHIs to address barriers posed by traditional clinical practice, these interventions have encountered low initiation rates, poor adherence over time, and high attrition [11-14]. Although previous studies have acknowledged these engagement concerns, few studies have assessed the types of barriers that may lead to DMHI disengagement. Early systematic reviews suggested that time constraints, perceived lack of treatment effectiveness, lack of motivation, and treatment burden were among the most frequently reported barriers to DMHI use [15,16]. A more recent review also found similar results highlighting the importance of user-level variables, such as help-seeking attitudes and perceived treatment fit and usefulness [17].

Part of the difficulty in understanding barriers that DMHIs users experience is the lack of standardized and validated measures. To date, most studies have relied on open-ended questions or ad hoc questionnaires that are specific to a given intervention or program [15-17]. Even when standardized measures of treatment barriers have been used in DMHIs trials [18], these scales were originally developed for traditional face-to-face therapy and did not capture the unique obstacles involved in using technology to receive care. For example, barriers in DMHIs could be related to the technological devices employed or the perceived lack of face-to-face support [17]. Identifying barriers commonly experienced in DMHIs will be fundamental to increasing enrollment and adherence, reducing attrition, and ultimately, improving outcomes.

This study addresses this research gap by developing the Digital Intervention Barriers Scale-7 (DIBS-7). Following a sequential QUAL → QUANT mixed methods design [19] and recommended procedures for measure development [20-22], we generated items based on feedback from DMHI users and refined them through DMHI expert review. Finally, we used factor analyses to establish the psychometric properties of this measure. This study design allowed findings at one stage to influence further methodological decisions, which is consistent with best practices in sequential mixed-methods research [19].

## Methods

### Recruitment

The data for this psychometric study came from an open trial [23] and a randomized controlled trial (RCT) [24] testing the effectiveness of a DMHI for young adults with depression and anxiety symptoms. This sample of treatment-seeking, young, and likely tech-savvy participants provided crucial information from individuals who, despite facing numerous barriers to DMHI treatment, were able to navigate these obstacles and complete treatment. Measure development consisted of 4 steps. In step 1, we used qualitative analyses to identify common barriers experienced by treatment completers in the open trial and generate items accordingly. In step 2, we reviewed previously developed validated measures of barriers in face-to-face therapy to develop relevant items, then a panel of DMHI experts reviewed the item pool and made a recommendation to ensure face validity and reduce item redundancy. In step 3, we conducted exploratory factor analyses (EFA) and confirmatory factor analyses (CFA) to determine the DIBS-7 structure and test its psychometric properties by administering this scale to treatment completers in the RCT. Lastly, in step 4, we estimated partial correlations to assess for criterion-related validity of the final version of the DIBS-7.

### Ethics Approval

All procedures in the open trial and RCT were approved by the Institutional Review Board of the University of California, Los Angeles (Protocol #17-000761), and were conducted in accordance with the Declaration of Helsinki and all other relevant guidelines and regulations. There were no protocol deviations in these studies. Participants provided written informed consent before beginning their participation, with the option to opt out of the study at any time without any type of penalty. Finally, data were deidentified before analyses to ensure participants’ privacy.

### Procedures

#### Step 1: Item Generation

After completing an open trial of a DMHI for anxiety and depression [23], 239 participants (mean age 22.9 years; 191/239, 80.3% female; 172/239, 72.1% racially or ethnically minoritized) provided feedback on their experience by answering open-ended questions related to barriers they faced while using the DMHI: “What was/were the biggest barrier(s) to completing the program? What would need to change to make this program better?” According to best practices in qualitative research [25], this sample size is adequate to achieve thematic saturation for qualitative analyses. Guided by thematic analysis [26], 2 coders familiar with the DMHI reviewed the user feedback to identify emergent themes related to barriers instead of defining themes a priori. Coders completed pilot training, received feedback, and double-coded all responses. Discrepancies and disagreements were resolved through weekly coding meetings with the master coder (LRRN). No missing data were present at this stage.

#### Step 2: DMHI Expert Review

To create new items in addition to those generated from the qualitative feedback in step 1, we reviewed previously validated and commonly used measures of barriers in traditional therapy. The scales examined were the Barriers to Treatment Participation Scale [27] and the Perceived Barriers to Psychotherapy [28]. Then, items generated in steps 1 and 2 were examined by DMHI experts with extensive knowledge in the development and implementation of DMHIs, including app-based programs, online interventions, and telehealth approaches to ensure item face validity and utility, examine measure format and response clarity, and eliminate redundancy [22]. The psychometric properties of the final item pool were evaluated in step 3.

#### Step 3: Psychometric Evaluation

Data for step 3 came from participants who completed a postintervention survey after participating in the RCT of the DMHI described in step 1 [24]. From over 1600 individuals who participated in the study, 559 completed the DIBS-7 (mean age 23.02 years). This sample was mostly female (438/559, 78.4%), racially or ethnically minoritized (374/559, 69.9%), and representative of the original sample, though there were slightly more females (*z*=2.18, *P*=.03). There were no other significant differences.

Considering evidence that factor analyses with ordinal and continuous data lead to virtually equivalent results when items have at least 5 response categories [29], all analyses in this study treated data as continuous. We examined data quality and factorability using Bartlett test of sphericity and the Kaiser-Meyer-Olkin (KMO) test. Data were randomly split into 2 groups: (1) EFA sample (n*=*200) and (2) CFA sample (n=359). According to scale development guidelines [20,21], these sample sizes provide sufficient statistical power to determine the factor structure of a 10-item scale using EFA while allocating more statistical power to confirm its factor structure and to compare it with alternative models using CFA. Following recommended practices in EFA [21], we conducted a principal axis factoring extraction method with an oblique Promax rotation in each EFA iteration. Eigenvalues and parallel analysis were used to determine the final number of factors to retain. Before confirming the final solution with CFA, we also examined potentially correlated residuals between items and modification indices to arrive at the best-fit solution.

After determining the final factor structure of the DIBS-7 and following guidelines in measure development [20-22], we calculated the internal reliability of the measure using Cronbach α and McDonald ω coefficients, considering α and ω values greater than 0.8 as acceptable.

#### Step 4: Validity Examination

Finally, using the same sample described in step 3, we estimated the initial criterion-related validity of the DIBS-7 by conducting partial correlations of its mean score with mean scores from previously validated measures of treatment expectations (ie, Treatment Motivation Questionnaire [TMQ]) [30] and treatment satisfaction (ie, Client Satisfaction Questionnaire [CSQ]) [31]. Similarly, the relationship between the DIBS-7 mean score and behavioral indicators of treatment adherence, including the number of modules with user activity and weekly user check-ins, was examined using the same approach. These analyses statistically controlled for user characteristics commonly associated with attitudes toward and perceptions of DMHIs and treatment engagement, such as user age, sex, and racially or ethnically minoritized status [15-17].

### Criterion-Related Validity Measures in Step 4

#### Treatment Expectations

The 5-item Confidence in Treatment subscale of the TMQ [30] was used to measure motivation for entering treatment. Participants rate items on a scale ranging from 1 (not at all true) to 7 (very true), with higher scores indicating higher motivation to receive treatment. Items include “I really want to make some changes in my life,” “I won't feel good about myself if I don't get some help,” and “It is important to me personally to solve my problems.” The TMQ has shown good reliability and has been associated with treatment adherence and dropout in previous studies [30]. In this study, the reliability of the Confidence in Treatment subscale was good (α=.87, ω=0.91).

#### Treatment Satisfaction

The 8-item CSQ [31] is a measure of satisfaction with treatment. Participants rate items on a scale ranging from 1 (poor; quite dissatisfied) to 4 (excellent; very satisfied), with higher scores indicating higher treatment satisfaction. Items include “How would you rate the quality of service you received?” “To what extent has our service met your needs?” and “How satisfied are you with the amount of help you received?” The CSQ has shown excellent reliability and has been associated with treatment adherence and dropout in previous studies [31]. In this study, the reliability of the CSQ was acceptable (α=.73, ω=0.79).

#### Treatment Adherence

The number of modules with user activity and weekly user check-ins were used as behavioral indicators of treatment adherence. Participants in the DMHI were required to log the number of skills practiced in each module every week. In addition to logging this information, participants were prompted to submit an end-of-week check-in with 2 reflective questions about skills practiced that week. Examples of reflective questions during weekly check-ins were “Which technique was most helpful for you?” and “Did this week move you closer or not to your goals?” Logging the number of modules with activity was mandatory, but submitting weekly check-ins was completely optional.

## Results

### Step 1: Item Generation

Results from the qualitative analyses indicated that a high percentage of participants mentioned at least 1 of 3 types of barriers: (1) difficulty remembering to use the intervention (79/239, 33%; eg, “I had a hard time remembering to do each challenge throughout the week”), (2) lack of self-motivation (67/239, 28%; eg, “I didn't feel accountable to do the tasks”), and (3) confusing or challenging intervention content (52/239, 21.7%; eg, “The program was confusing. It didn't provide the level of challenges I expected”). These qualitative results highlighted the importance of including items capturing both user-level (eg, motivation) and DMHI-level (eg, content) barriers in developing the DIBS-7. For instance, item 4 (ie, “I forgot to use the DMHI”) was created to capture the difficulty of remembering to engage in a DMHI. Similarly, item 5 (ie, “It was difficult to keep myself motivated to use the DMHI”) was developed to measure motivational challenges to adhere to a DMHI. Finally, item 2 (ie, “I didn’t understand the tasks or things I was supposed to do in the DMHI”) and item 3 (ie, “I thought the DMHI wasn’t engaging”)* *were created to capture barriers related to perceptions of DMHI content.

### Step 2: DMHI Expert Review

Following DMHI experts’ recommendations, we examined the Barriers to Treatment Participation Scale [27] and the Perceived Barriers to Psychotherapy [28] to generate additional items. Although these scales focus on barriers encountered in traditional face-to-face services, this process facilitated the creation of items related to (1) treatment content and demands (eg, item 6: “I thought the length of the DMHI wasn’t adequate [ie, too long or too short]”), (2) perceived relevance or efficacy of the intervention (eg, item 6: “The DMHI was available when I needed it”; item 7: “I felt that I needed support from a therapist and not just from a DMHI”; item 9: “I felt the DMHI did not focus on my life and problems”; item 10: “The DMHI did not seem to be helping me”). Finally, as suggested by DMHI reviewers, we created items related to technology-based obstacles to capture unique barriers associated with DMHIs (eg, item 1: “I had technical problems with my technology [eg, device, internet, website]”).

The same 2 DMHI experts examined the final item pool to ensure item face validity and utility, measure format, response clarity, and eliminate redundancy. In response to these experts’ feedback, we consolidated similar items (eg*,* “The DMHI was confusing/The DMHI did not have a goal” *→* “I did not understand the tasks or things that I was supposed to do in the DMHI”) and eliminated additional items deemed redundant (eg, “I did not want to practice the DMHI tasks”) or lacking face validity (eg, “I would have a hard time finding information on the internet”) leading to a final item pool of 10. We administered these 10 items for validation purposes. Table 1 shows items tested during the validation of the DIBS-7, means, standard deviations, and factor loadings.

**Table 1:** Digital Intervention Barriers Scale-7 (DIBS-7) items evaluated, means, SDs, and exploratory factor analysis (EFA) factor loadings.

Item	Mean (SD)	EFA^a^ factor loading
1. I had technical problems with my technology (eg, device, internet, platform)	1.51 (1.01)	0.75
2. I didn’t understand the tasks or things I was supposed to do in the DMHI^b^	1.88 (1.18)	0.65
3. I thought the DMHI wasn’t engaging	2.33 (1.16)	0.75
4. I forgot to use the DMHI	3.32 (1.42)	0.67
5. It was difficult to keep myself motivated to use the DMHI	3.25 (1.39)	0.75
6. The DMHI was available when I needed it^c^	1.08 (1.10)	0.09
7^c^. I felt I needed additional support from a therapist and not just from a DMHI^c^	1.59 (1.41)	0.36
8. I thought the length of the DMHI wasn’t adequate (too long or too short)	2.41 (1.08)	0.58
9. I felt the DMHI did not focus on my life and problems^c^	2.27 (1.13)	0.15
10. The DMHI did not seem to be helping me	2.63 (1.19)	0.79

^a^EFA factor loadings reported are based on a 1-factor solution.

^b^DMHI: digital mental health intervention.

^c^Item excluded from the final version of the DIBS-7.

### Step 3: Psychometric Evaluation

Bartlett test of sphericity was significant (*χ*^2^_45_=637.77, *P*<.001), and the KMO measure was middling (KMO=0.79) [32]. In an EFA including all 10 items, eigenvalues, and parallel analysis suggested a factor solution ranging from 1 to 3 factors. Thus, all 3 solutions (ie, 1-factor, 2-factor, 3-factor) were fitted independently and evaluated based on fit indices (ie, model *χ*^2^, standardized root mean square residual [SRMR], Tucker Lewis Index [TLI], root mean square error of approximation [RMSEA]).

A 1-factor solution explained 32.26% variance overall. Based on a 0.5 loading cutoff value to maximize the reliability of factor recovery, items 6 (0.09), 7 (0.36), and 9 (0.15) were inadequate. Indices suggested some fit problems (*χ*^2^_35_=157.05, *P*<.001; RMSR=0.09; TLI=0.73; RMSEA=0.13). A 2-factor solution explained 66% variance overall. This solution led to a factor composed of items 3 (0.61), 4 (0.73), 5 (0.92), 8 (0.53), and 10 (0.76), and another factor composed of items 1 (0.75) and 2 (0.77). Items 6 (–0.07, 0.22), 7(0.25, 0.16), and 9 (–0.01, 0.23) did not load adequately in any of these 2 factors. Fit indices were similar to those of the 1-factor model (*χ*^2^_26_=72.69, *P*<.001; RMSR=0.06; TLI=0.86; RMSEA=0.095). A 3-factor solution explained 58% variance overall. Loadings suggested 1 factor composed of items 3 (0.59), 4 (0.71), 5 (0.91), 8 (0.54), and 10 (0.77), another factor with items 1 (0.77) and 2 (0.72), and a final factor composed of items 7 (0.58) and 9 (0.35). Items 6 (–0.04, 0.26, –0.10), 7 (0.12, –0.04, 0.58), and 9 (–0.17, 0.07, 0.45) did not load adequately in any of these 3 factors. Fit indices were similar to those of previous models (*χ*^2^_18_=54.98, *P*<.001; RMSR=0.04; TLI=0.84; RMSEA=0.1).

Given that items 6, 7, and 9 did not seem to load adequately in any of the 3 models analyzed, we dropped these items and reestimated potential factor solutions using the same sample. Bartlett test of sphericity was significant (*χ*^2^_21_=574, *P*<.001) and the KMO measure was meritorious (KMO=0.80). In an EFA with 7 items, eigenvalues and parallel analysis suggested 1 or 2 potential factors. A 1-factor solution explained 43.95% variance overall. All items loaded above the 0.50 cutoff value except for item 1 (0.32). Indices suggested some fit issues (*χ*^2^_14_=114.5, *P*<.001; RMSR=0.1; TLI=0.73; RMSEA=0.19). A 2-factor solution explained 66% variance overall. This solution led to a factor composed of items 3 (0.59), 4 (0.74), 5 (0.90), 8 (0.51), and 10 (0.74), and another factor composed of items 1 (0.73) and 2 (0.81). Fit indices were similar to those of the 1-factor model (*χ*^2^_8_=39.1, *P*<.001; RMSR=0.05; TLI=0.85; RMSEA=0.14).

As a whole, EFA results seemed to suggest that a 1-factor model was the most acceptable solution. Considering that items 1 and 2 appeared to be related to one another in several models, we fit a 1-factor solution using the exploratory data to allow for correlated residuals between these items and examine modification indices that could improve model fit. The solution with correlated residuals between items 1 and 2 still presented some fit problems (*χ*^2^_13_=54.39, *P<*.001; comparative fit index [CFI]=0.927, TLI=0.882, RMSEA=0.126, SRMR=0.052). Modification indices also suggested a correlated residual between items 4 and 5 (modification index=44.089). As such, we fit a 1-factor solution allowing correlated residuals between items 1 and 2 as well as 4 and 5. This model led to a satisfactory solution (*χ*^2^_12_=31.01, *P<*.001; CFI=0.979, TLI=0.962, RMSEA=0.066, SRMR=0.030). We directly compared models with correlated residuals between items 1 and 2 versus a model with no correlated residuals. The model with correlated residuals was a better fit for the data (*χ*^2^_1_=61.52, *P*<.001); then, we compared this model against one with correlated residuals between items 1 and 2 as well as items 4 and 5. Results indicated that the model with both correlated residuals was a more satisfactory solution (*χ*^2^_1_=38.72, *P*<.001).

Finally, using the confirmatory data, we fit the best 2 competing models (ie, 1-factor solution with correlated residuals between 1 and 2 vs 1-factor solution with correlated residuals between items 1 and 2 as well as 4 and 5) using CFA analyses. Then, we directly compared both models to determine the best factor solution. Results indicated that the solution with 2 correlated residuals led to a more satisfactory solution compared with the solution with only 1 correlated residual (*χ*^2^_1_=50.56, *P*<.001). The model with 2 correlated residuals showed better fit indices (*χ*^2^_12_=31.01, *P<*.001; CFI=0.979, TLI=0.962, RMSEA=0.066, SRMR=0.030) compared to the model with only 1 correlated residual (*χ*^2^_13_=81.57, *P<*.001; CFI=0.923, TLI=0.875, RMSEA=0.121, SRMR=0.050). The final CFA solution consisted of 1 factor with 7 items with a high internal consistency (α=.82, ω=0.89).

### Step 4: Validity Examination

Following best practices to determine the initial criterion-related validity of new measures [22] and using the full sample (*n=*559), we estimated partial correlations between the mean score of the 7-item DIBS and previously validated measures of treatment satisfaction (ie, TMQ) and expectations(ie, CSQ) as well as behavioral indicators of treatment engagement (ie, logged activities, weekly check-ins), while statistically controlling for user characteristics that have shown to be related to DMHI treatment engagement, including age, sex, and racial or ethnic minoritized status [15-17]. Initial criterion-related validity of the measure was supported by significant partial correlations between the DIBS-7 mean score and treatment expectations (*pr*_553_=–0.250, *P<*.001), number of weekly user check-ins (*pr*_553_=–0.282, *P<*.001), number of modules with user activity (*pr*_553_=–0.556, *P<*.001), and treatment satisfaction (*r*_553_=–0.714, *P<*.001). See Multimedia Appendix 1 for the final version of the DIBS-7.

## Discussion

### Principal Findings

Preliminary evidence suggests the DIBS-7 is a valid measure of DMHI barriers. Using a sequential mixed-methods design, we were able to identify 7 items that capture commonly faced barriers in DMHIs. Qualitative analyses of DMHI users’ feedback and DMHI expert review facilitated the creation of items showing content-related validity. Statistical analyses determined the structural validity of the scale, revealing a unidimensional measure with excellent internal consistency. Further, preliminary criterion-related validity was established by finding convergence between the DIBS-7 mean score and well-established measures of related constructs, such as treatment expectations, behavioral indicators of treatment engagement, and treatment satisfaction, after statistically controlling for user characteristics associated with DMHI treatment engagement, such as participant’s age, sex, and racially or ethnically minoritized status. This methodology is consistent with gold-standard procedures in measure development [20-22] and mixed-methods research [19], a strength of this study. As such, the DIBS-7 represents a promising scale of treatment barriers in DMHIs.

Despite being developed using a specific type of DMHI (ie, self-guided, web-based cognitive behavioral therapy intervention for anxiety and depression), the final content of the DIBS-7 seems to map onto both user-level (eg, beliefs, attitudes) and intervention-level (eg, type of content, technology used) factors common across different DMHI modalities [15-17]. While barriers in DMHIs can be numerous and unique to each type of modality (eg, self-guided vs therapist-supported), a broad, yet psychometrically sound scale may contribute to improving DMHI clinical practice and research. For instance, routine monitoring of barriers can facilitate the identification of users who need additional support to engage in DMHIs [33]. Similarly, in research, tracking DMHI barriers is fundamental to assess how well the intervention is being implemented against expected results at any stage of DMHI development, including early prototypes or pilots, efficacy and effectiveness trials, and scaling-up and sustainability studies [34]. Thus, the DIBS-7 can address a need in the DMHI field by providing clinicians and researchers with a short, standardized, and validated measure that potentially reduces time concerns and patient burden, which are significant obstacles to evidence-based outcome monitoring [33,35].

Another factor supporting the potential utility of the DIBS-7 as a short measure of barriers in DMHIs is its criterion-related validity. The DIBS-7 mean score significantly converged with well-established variables associated with DMHI barriers, such as treatment expectations, behavioral indicators of treatment engagement, and treatment satisfaction [15-17]. Importantly, these associations were significant over and above user characteristics that have been shown to be related to treatment engagement in DMHI trials [15,17]. Thus, this measure may be useful for a wide range of DMHI users regardless of their age, sex, or racial and ethnic identity. Further supporting the validity of the DIBS-7, in the DMHI examined in this study, the number of weekly user check-ins and modules with user activity was associated with symptom reductions [36]. Similarly, treatment expectations were also a significant moderator of treatment response [24]. Therefore, the DIBS-7 is associated with variables that directly impact the effectiveness of DMHIs and the magnitude of their treatment effects.

### Limitations and Future Directions

Despite the implications of having a short and validated measure of DMHI barriers, the results of this study need to be examined considering certain limitations. For instance, correlated residual among items 1 and 2 as well as 4 and 5 may suggest the DIBS-7 could benefit from having subscales that capture related but somewhat different aspects of DMHI barriers (eg, issues with technology, attitudinal factors, desire for human support). Although current findings support using the DIBS-7 as a reliable and valid measure of barriers in DMHIs, additional research with this measure could help further refine its items and identify new subscales needed, which may lead to a broader assessment of different types of barriers and improved psychometric properties. Further, the content of the DIBS-7 was intentionally broad to be applicable across different types of DMHIs. However, user feedback guiding the development of scale items was based on users’ experience in a specific type of DMHI (ie, self-guided, web-based cognitive behavioral therapy program). As such, data capturing the experience of users in other DMHI modalities (eg, app-based programs, coach-assisted interventions, acceptance-based treatments) who may face other types of barriers are needed. Whether specific measures for each type of DMHI are required or broad scales are adequate is still an empirical question that requires further examination. Therefore, future studies are needed to determine whether the DIBS-7 is a reliable and valid measure across different types of DMHIs. Finally, participants in this study included treatment-seeking, college-aged, likely tech-savvy, English-speaking individuals who were able to complete a self-guided DMHI. Arguably, this group is among the least impacted by DMHI barriers. Accordingly, additional efforts are needed to include community samples that often experience significant and unique barriers when trying to benefit from DMHIs, including being a member of one or more marginalized groups, having fewer and older technological devices at home, limited access to the Internet, low tech literacy, and dealing with DMHI content that is not culturally or contextually relevant [6,37]. Indeed, future studies with more diverse samples that include younger and older individuals, members of minoritized groups beyond race and ethnicity, nontreatment completers, persons from low-socioeconomic status, and non-English speakers will facilitate the refinement of the DIBS-7 and establish its validity across groups with socially complex needs.

### Conclusions

With the growing interest in and use of DMHIs, understanding common barriers faced by users is crucial for improving treatment adherence and outcomes in these interventions. The DIBS-7 addresses a need in the field by providing a short and psychometrically sound measure of barriers in DMHIs. This scale represents a valuable tool that can be easily implemented in routine clinical care and DMHI research. Significantly, findings from this study may increase interest in developing more comprehensive measures that capture the experience of a wide range of DMHI users.

## Appendix

Multimedia Appendix 1 Digital Intervention Barriers Scale–7 (DIBS-7).

## Abbreviations

CFA: confirmatory factor analyses
CFI: comparative fit index
CSQ: Client Satisfaction Questionnaire
DIBS-7: Digital Intervention Barriers Scale-7
DMHI: digital mental health intervention
EFA: exploratory factor analyses
KMO: Kaiser-Meyer-Olkin
RCT: randomized controlled trial
RMSEA: root mean square error of approximation
SRMR: standardized root mean square residual
TLI: Tucker Lewis index
TMQ: Treatment Motivation Questionnaire
