# Supplementary material for: Digital Intervention Barriers Scale–7 (DIBS-7): Development, Evaluation, and Preliminary Validation
Source: JMIR Form Res. 2023 Apr 6;7:e40509. doi: 10.2196/40509 (PMC10131680; doi:10.2196/40509)
Supplement: Multimedia Appendix 1 [file formative_v7i1e40509_app1.docx]

**Appendix.**

**Digital Intervention Barriers Scale–7 (DIBS-7)**

Please indicate the extent to which you agree or disagree with the following statements

1. *I had technical problems with my technology (device, internet, platform)*

*Totally Disagree 1 2 3 4 5 Totally Agree*

1. *I didn’t understand the tasks or things I was supposed to do in the DMHI*

*Totally Disagree 1 2 3 4 5 Totally Agree*

1. *I thought the DMHI wasn’t engaging*

*Totally Disagree 1 2 3 4 5 Totally Agree*

1. *I forgot to use the DMHI*

*Totally Disagree 1 2 3 4 5 Totally Agree*

1. *It was difficult to keep myself motivated to use the DMHI*

*Totally Disagree 1 2 3 4 5 Totally Agree*

1. *I thought the length of the DMHI wasn’t adequate (too long or too short)*

*Totally Disagree 1 2 3 4 5 Totally Agree*

1. *The DMHI did not seem to be helping me*

*Totally Disagree 1 2 3 4 5 Totally Agree*
